# Another One

**Published:** 1885-12

**Authors:** 


### ANOTHER ONE.

DR. AUSTIN FLINT, Sr., comes forward with the suggestion that acute coryza is a parasitic disease, and predicts that the germ upon which it depends will soon be discovered. In an article in the *Jour. of the Amr. Med. Association* he brings forward arguments in support of this view, and recommends treatment of the disease by topical application of germicides; if applications of this kind should prove succesful, Dr. Flint thinks the parasitic origin of the disease would be partially proved in advance of the discovery of the parasite.

The germ theory has become such a mighty war that it promises to swallow up all diseases in its depths. In view of the number of diseases which have been assigned an especial microbe it would be but a small step to claim that every form of disease is of parasitic origin. Nevertheless we believe that coryza will be a last ditch for the anti-bacterial pathologists, and that it will require little less than a revolution to overthrow the faith of laity and doctors in that time-honored explanation of all diseases, "taking cold."—*N. Y. Lancet.*

